# Unstable Spinal Fracture With Arteria Lusoria: Management to Mitigate Aortic Risks

**DOI:** 10.7759/cureus.69025

**Published:** 2024-09-09

**Authors:** Aziza Kantri, Khalid Agrad, Najwa Benslima, Fadwa Fliyou, Afak Nsiri, Mustapha Bensghir, Hicham Bakkali, Chafik Elkettani

**Affiliations:** 1 Anesthesia and Critical Care, Mohammed VI International University Hospital, Faculty of Medicine, Mohammed VI University of Health Sciences, Casablanca, MAR; 2 Radiology, Mohammed VI University of Health Sciences, Casablanca, MAR; 3 Neurosurgery, Cheikh Khalifa International University Hospital, Mohammed VI University of Health Sciences, Casablanca, MAR; 4 Anesthesiology and Critical Care, Ibn Rochd University Hospital, Faculty of Medicine and Pharmacy, Hassan II University, Casablanca, MAR; 5 Anesthesia and Critical Care, Mohammed V Military Teaching Hospital, Mohammed V University, Rabat, MAR; 6 Anesthesia and Critical Care, Mohammed V Military Training Hospital, Faculty of Medicine and Pharmacy, Mohammed V University, Rabat, MAR; 7 Anesthesiology and Critical Care, Faculty of Medicine and Pharmacy, Hassan II University, Casablanca, MAR

**Keywords:** arteria lusoria, blunt traumatic aortic injuries, endovascular procedure, spinal fracture, spine stabilization

## Abstract

Aortic injuries associated with unstable spinal fractures are a rare but serious condition, with high mortality. Rapid and multidisciplinary management is crucial to prevent fatal complications. We report the case of a female patient who, following a road traffic accident, presented with a displaced fracture of the fourth dorsal vertebrae (D4), with a detached anterior fragment adjacent to the posterior aspect of the aortic arch and the origin of the arteria lusoria. The multidisciplinary discussion concluded that surgery without an aortic prosthesis is associated with a major hemorrhagic risk due to aortic injury during spinal fixation manipulations, and surgery with a prosthesis is associated with immediate risks of ischemic cerebrovascular accident, gas embolism, and upper limb ischemia. We opted to prepare the patient for spinal stabilization surgery after placing the aortic prosthesis type: Zenith Alpha ZTA-PT-30-26-108 thoracic prosthesis (Cook Medical, Bloomington, Indiana, US). Our team's therapeutic approach is being discussed given the rarity of cases in the literature and the patient's anatomical characteristics. Surgical management in these situations must repair the unstable fracture while avoiding the aggravation of an existing or potential aortic injury. The aortic lesion can be treated first, before spinal fixation, either with open surgery, which carries a high risk of severe complications, or thoracic endovascular repair (TEVAR), which allows the prevention of the potential aortic injury or repair of the existing aortic injury while minimizing the side effects of open surgery. However, endovascular surgery may have limitations due to individual vascular anatomy, as in our patient’s case, which can prevent optimal endograft positioning and lead to risks such as endoleak, ischemia, infection, or thrombosis, necessitating periodic radiological follow-up. Endovascular repair is a new paradigm that has improved clinical outcomes for these patients by securing the vascular injury first before spinal surgery. Teamwork and multidisciplinary discussion ensure optimal safety, minimizing the side effects of these lesions, which can be fatal at any time during management.

## Introduction

Concomitant aortic injuries with unstable spinal fractures are a rare occurrence but are associated with a high mortality rate exceeding 85% of cases [1-3]. These injuries are secondary to high-energy trauma, applying direct forces that compress the aorta through the spinal column. When the vertebrae are fractured by these forces, the resulting bone fragments can lacerate the aorta [4], either spontaneously or during surgical osteosynthesis. Managing the vascular injury is a priority and should be performed before spinal surgery [4-5]. If spinal surgery is performed first, mortality can reach 60%. This can be managed either through open surgery, which carries significant risks of complications, or TEVAR, which allows for the prevention of the potential aortic injury or repair of an existing aortic injury while minimizing the side effects of open surgery. However, endovascular surgery may have limitations due to individual vascular anatomy, which can prevent optimal endograft positioning and lead to risks such as endoleak, infection, or thrombosis, necessitating periodic radiological follow-up [6-7].

We report the case of a patient with a displaced fracture of the D4, with a detached anterior fragment adjacent to the posterior aspect of the aortic arch and the origin of the arteria lusoria. We propose a discussion of the therapeutic approach in our patient, given the rarity of cases in the literature and the anatomical variation present.

## Case presentation

**Figure 1:**
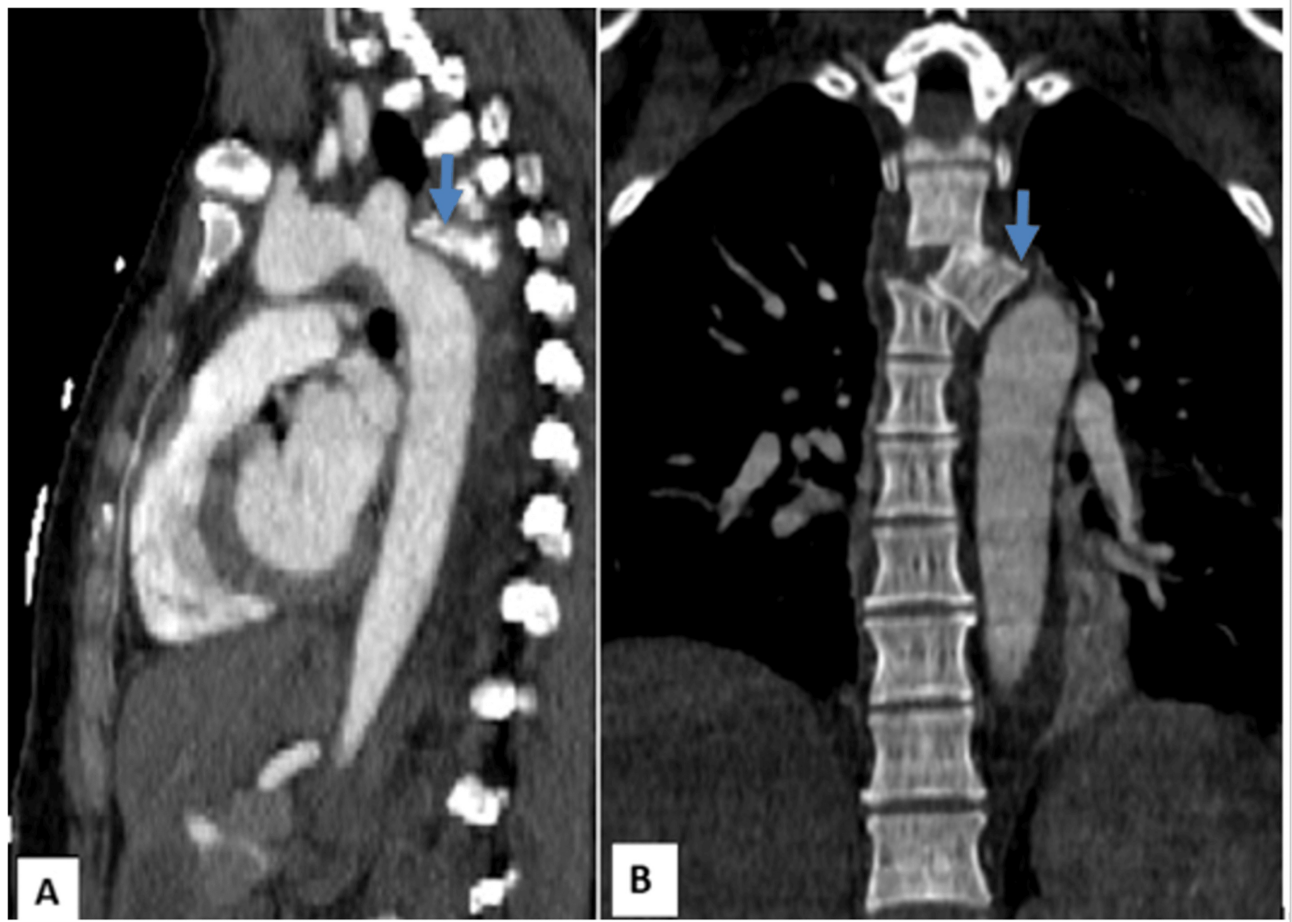
Sagittal (A) and coronal (B) CT angiography Displaced fracture in the left paravertebral region of the fourth dorsal vertebrae (blue arrow), encroaching the descending thoracic aorta although without signs of wall disruption (i.e., absent peri-vascular contrast leakage)

**Figure 2:**
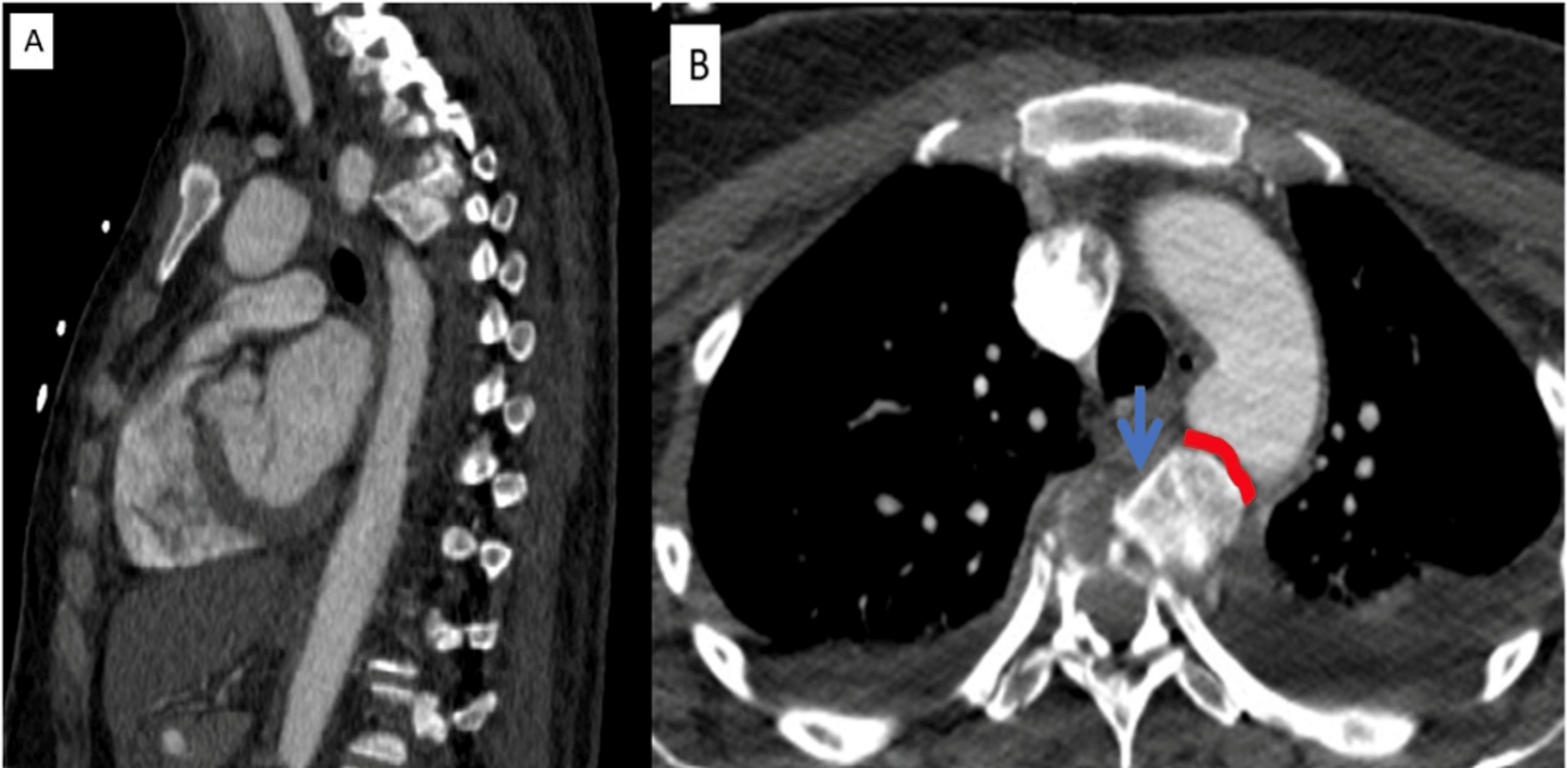
Sagittal (A) and axial (B) CT angiography Compression fracture with left anterolateral displacement of the fourth dorsal vertebrae (blue arrow), encroaching the posterior side of the aortic arch and the origin of the right subclavian artery, with a maintained rim of separation (red line)

**Figure 3:**
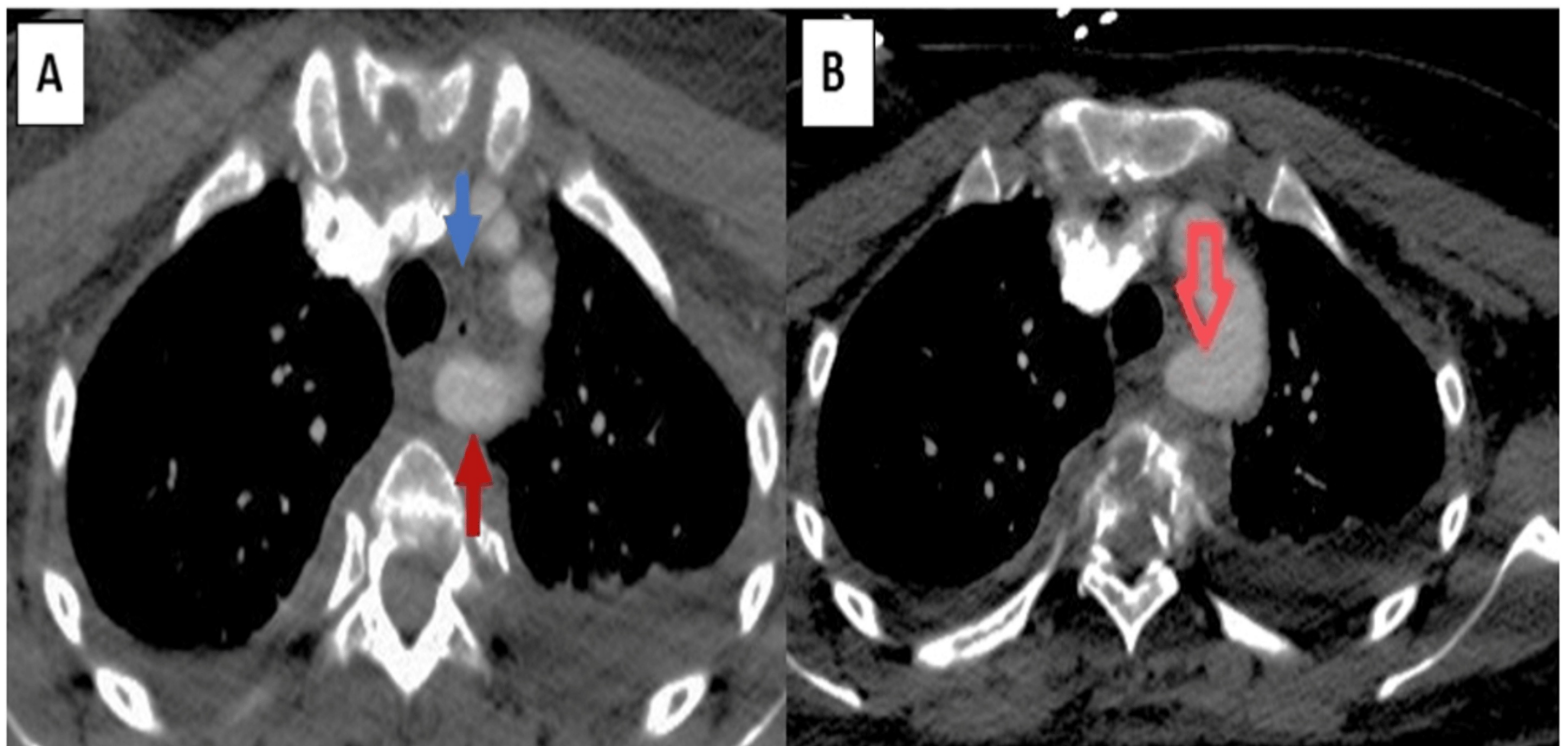
Axial (A and B) CT angiography A right subclavian artery (lusoria) (solid red arrow) arising from a Kommerell diverticulum (red arrow) at the level of the bone fragment compressing the aortic arch with no signs of esophageal or tracheal compression (blue arrow). The left subclavian artery originates from the anterolateral side of the aortic arch in front of the origin of the right subclavian artery. The common carotid arteries on the right and left are unremarkable.

**Figure 4:**
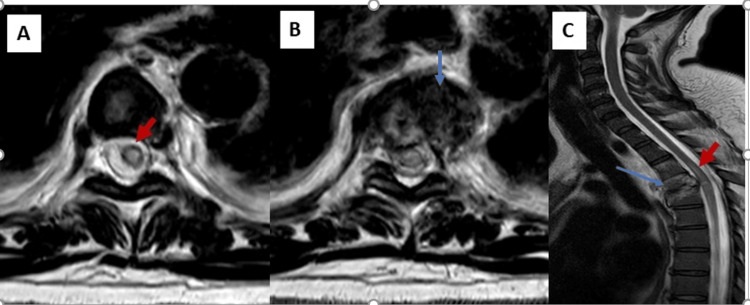
MRI T2 sequence in axial (A, B) and sagittal (C) views Displaced compression fracture of the fourth dorsal vertebrae on the left paravertebral side (blue arrow), causing posterior wall retraction, reducing the subarachnoid spaces, and compressing the adjacent spinal cord, with T2 hyperintensity indicating a spinal cord injury (red arrow)

A 43-year-old female patient, with no significant medical history, was admitted to our facility for the management of a vertebro-medullary trauma following a high-kinetic energy public road accident. Upon admission, the patient was conscious with stable hemodynamic and respiratory conditions, paraplegic with a sensory level at D4. The lower limb deep tendon reflexes were absent, the Babinski reflex was equivocal, and there was no pyramidal syndrome. On rectal examination, there was anal laxity, and the patient also presented with non-active otorrhagia on the left side and a sutured left frontal wound. A body CT scan revealed a pulmonary contusion focus with bilateral pleural effusion of low volume, a displaced fracture of the D4 with a detached anterior fragment adjacent to the posterior aspect of the aortic arch and the origin of the right retroesophageal subclavian artery, responsible for a prevertebral space hematoma, a fracture of the right posterior lamina with intracanal fragments, fracture of the anterior-superior corner with a detached fragment and the right transverse process of D5, fracture of the right transverse process of D3, L2, L3, and the sternal manubrium (Figure 1). A thoracic computed tomography angiography (CTA) showed a compression fracture and anterolateral left slip of D4 adjacent to the posterior aspect of the aortic arch and the origin of the right retroesophageal subclavian artery (arteria lusoria), with a maintained separating line (Figures 2-3). Spinal MRI showed a compression fracture of the D4 with posterior wall displacement and signs of spinal cord distress at D4-D5 (Figure 4).

A multidisciplinary discussion including critical care physicians, vascular surgeons, radiologists, neurosurgeons, and rehabilitation physicians concluded that surgery without an aortic prosthesis is associated with a major hemorrhagic risk due to aortic injury during spinal fixation manipulations, and surgery with a prosthesis is associated with immediate risks of ischemic cerebrovascular accident (ICVA), gas embolism, and upper limb ischemia. We opted to prepare the patient for spinal stabilization surgery after placing the aortic prosthesis.

An anatomical assessment for the prosthesis placement was performed with a second thoracic CT angiography, revealing a right subclavian artery (lusoria) originating from a Kommerell diverticulum at the base of the bone fragment compressing the aortic arch without signs of esophageal or tracheal compression, with the left subclavian artery originating from the anterolateral aspect of the aortic arch facing the origin of the right subclavian artery, with no abnormalities in the right and left common carotid arteries. In the operating room, the patient underwent the placement of a Zenith Alpha ZTA-PT-30-26-108 thoracic prosthesis graft (Cook Medical, Bloomington, Indiana, US) under fluoroscopic control with release at the base of the left subclavian artery and expansion of the prosthesis with a Coda balloon. Aortography confirmed the proper positioning and adjustment of the endoprosthesis, with patent vascular branches. Immediately after the placement of the aortic prosthesis, the neurosurgery team stabilized the dorsal spine with posterior fixation and pedicle screw placement at D3, D5, and D6 and dorsal rod placement with fluoroscopic guidance. The surgery proceeded without particular incidents except for some hemodynamic changes controlled by deepening anesthesia, vascular filling, and low doses of vasoconstrictors.

**Figure 5:**
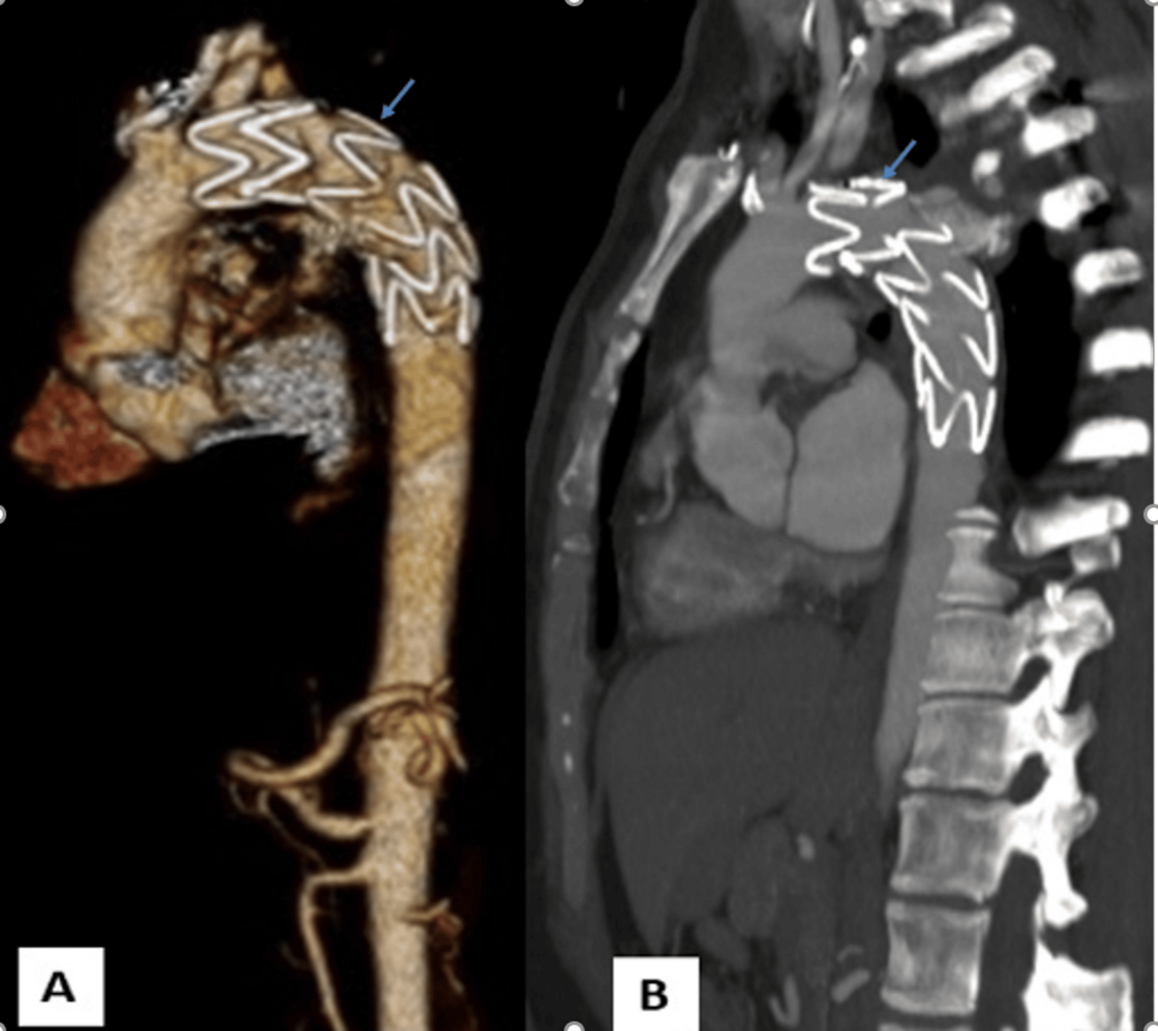
Post-treatment CT-angiography 3D reconstructions views (A) and sagittal (B): aortic stent-graft in place (blue arrow)

**Figure 6:**
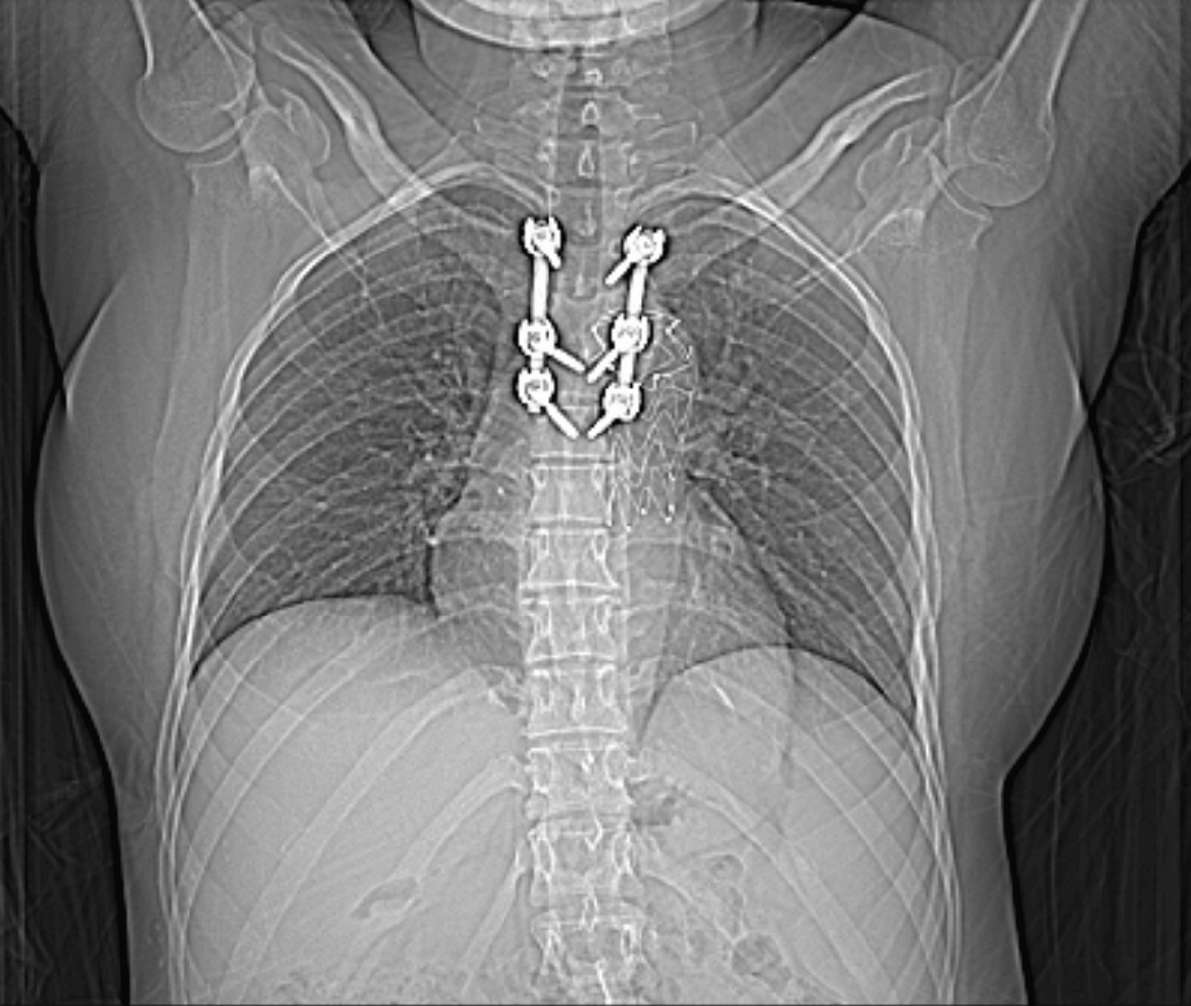
CT scan Aortic stent-graft in place with posterior instrumentation of the spine

The assessment was completed with a thoracic CT angiography showing good aortic opacification with the prosthesis in place without contrast product extravasation, and good opacification of the right retroesophageal subclavian artery without parietal hematoma. Postoperatively, the patient remained stable, she had partially to fully recovered her paraplegia, was put on antiplatelet therapy, and was discharged with a physical rehabilitation protocol. Follow-up imaging at one month showed satisfactory alignment of the spine and the placement of the hardware and no complications related to the prosthesis (Figures 5-6).

## Discussion

In non-penetrating thoracic trauma, the combination of aortic injury and spinal fracture is rare but possible and is described in approximately 5.5% of cases; this incidence is likely underestimated [8] given the number of deaths occurring at the scene of the accident without medical care, with an out-of-hospital mortality rate of 75% to 85% [5]. These aortic injuries can be primary, resulting from distraction dislocation forces frequently observed in type C fractures, or secondary to manipulations during spinal surgery. Several studies have described iatrogenic aortic injuries after spinal surgery [8-13] while few report the management of unstable spinal fractures with concomitant or potential aortic injuries [8,14-16]. Regardless of the direct or secondary origin, the consequences can be disastrous, with imminent hemorrhagic risk requiring specific and multidisciplinary management. Carlotta et al., in a retrospective analysis of all patients diagnosed with blunt traumatic aortic injuries (BTAIs) from January 1999 to January 2020, concluded that TEVAR is becoming the main treatment line. This is in line with the 2011 clinical practice guidelines of the Society for Vascular Surgery (SVS) for the management of BTAIs, which recommend TEVAR over open repair, given better survival rates and lower paraplegia rates [17].

These studies have confirmed that open surgery via thoracotomy is associated with a relatively high morbidity rate such as impaired ventilatory function with the risk of severe pneumonia that can develop into acute respiratory distress syndrome (ARDS), renal failure, and spinal cord ischemic lesions with the risk of permanent sequelae. Additionally, these patients often present with traumatic lung injuries contraindicating pulmonary exclusion during perioperative ventilation [2], and the lateral decubitus position required for thoracotomy may exacerbate spinal instability, leading to spinal cord injury [1-12].

To address these constraints, a paradigm shift in the management of BTAIs has been adopted in recent years in favor of TEVAR, as it is less invasive: the procedure is short, performed in the supine position, and does not involve vascular clamping, reducing the risk of respiratory complications and paraplegia. Although the superiority of TEVAR has been established, controversy still surrounds certain aspects, including the optimal timing of the intervention, individual vascular anatomical peculiarities preventing optimal endoprosthesis positioning, placing aortic branches at risk or favoring graft oversizing with distal migration, folding, and/or thrombosis [18-19], with the use of antiplatelet agents.

Thus, a thorough evaluation of these anatomical variations that can influence this vascular repair is essential through relevant imaging: CT +/- conventional angiography and MRI. In our case, the patient presented with an arteria lusoria, the most common aortic arch anomaly, occurring in 0.5% to 2.5% of individuals, with the origin of the four vessels sequentially from the aortic arch: the right common carotid artery, left common carotid artery, left subclavian artery, and aberrant right subclavian artery [19]. This anomaly is usually asymptomatic, often discovered incidentally during the evaluation of other mediastinal lesions or clinically manifested by dysphagia due to esophageal compression. The coexistence of this anomaly in our patient raised concerns about ischemic or maldimensioning risk after the placement of the aortic prosthesis. Teamwork and multidisciplinary discussion ensure optimal safety, minimizing the side effects of these lesions, which can be fatal at any time during management.

## Conclusions

Concomitant or potential aortic injuries associated with spinal fractures, though rare, are fatal. They can present acutely or develop with pseudoaneurysm formation and delayed bleeding. Their detection should be systematic in severe or high-energy trauma through relevant imaging, which also allows for the detection of associated anomalies that may compromise the management of these patients. Endovascular repair is a new paradigm that has improved clinical outcomes for these patients by securing the vascular injury first before spinal surgery. Finally, multidisciplinary teamwork is the key to success in this management.
